# Hierarchical Design in Nanoporous Metals

**DOI:** 10.1002/advs.202106117

**Published:** 2022-07-28

**Authors:** Jie Ying, Silvia Lenaerts, Mark D. Symes, Xiao‐Yu Yang

**Affiliations:** ^1^ School of Chemical Engineering and Technology Sun Yat‐sen University (SYSU) Zhuhai 519082 P. R. China; ^2^ State Key Laboratory of Advanced Technology for Materials Synthesis and Processing Wuhan University of Technology Wuhan 430070 P. R. China; ^3^ School of Engineering and Applied Sciences Harvard University Cambridge MA 02138 USA; ^4^ Research Group of Sustainable Energy and Air Purification (DuEL), Department of Bioscience Engineering University of Antwerp Groenenborgerlaan 171 Antwerp 2020 Belgium; ^5^ WestCHEM, School of Chemistry University of Glasgow Glasgow G12 8QQ United Kingdom

**Keywords:** electrochemical applications, hierarchical design, nanoporous metals, structure–function relationship, synthetic strategies

## Abstract

Hierarchically porous metals possess intriguing high accessibility of matter molecules and unique continuous metallic frameworks, as well as a high level of exposed active atoms. High rates of diffusion and fast energy transfer have been important and challenging goals of hierarchical design and porosity control with nanostructured metals. This review aims to summarize recent important progress toward the development of hierarchically porous metals, with special emphasis on synthetic strategies, hierarchical design in structure–function and corresponding applications. The current challenges and future prospects in this field are also discussed.

## Graphical abstract

As a new group of hierarchical materials, hierarchically porous metals not only present high accessibility of matter molecules, but also provide unique continuous metallic frameworks for greatly enhancing electron mobility and utilization. This review summarizes the most commonly used synthesis strategies to produce hierarchically porous metals and discusses how this hierarchy impacts on structure–function for applications in catalysis, energy, and sensing.

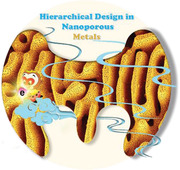

## 1 Introduction

Hierarchically porous materials are attracting considerable interest due to their potential applications in catalysis, separation, optics, energy and life science.^[^ ^1^, ^2^, ^3^, ^4^, ^5^, ^6^, ^7^, ^8^, ^9^, ^10^, ^11^ ^]^ They present structures with a range of microporosity (<2 nm), mesoporosity (2–50 nm), and macroporosity (>50 nm), with their exceptional properties, often held to be ascribed to this structural hierarchy. For example, the introduction of mesoporosity into microporous materials minimizes diffusion limitations in liquid‐phase catalysis, and thus enhances catalytic activity.^[^ ^12^, ^13^ ^]^

Control of hierarchical porosity has emerged as a way to significantly enhance the performance of nanostructured metals for applications in catalysis and electrocatalysis, as well as a way to improve catalyst stability in various processes. In particular, hierarchical design in porous structures has been central to driving the development of nanostructured metals. As a new group of hierarchical materials, hierarchically porous metals not only present high accessibility of matter molecules, but also provide unique continuous metallic frameworks for greatly enhancing electron mobility and utilization.^[^ ^14^, ^15^ ^]^ These advantageous features share a core requirement of high surface energy, which directly leads to a high level of exposed active atoms. Further considering practical applications such as catalysis, high rates of diffusion and fast energy transfer have been important and challenging goals of hierarchical design and porosity control with nanostructured metals. In the past few decades, a great number of advanced synthesis strategies have been developed to rationally design and regulate the pore sizes and structures of hierarchically porous metals (**Table** **1**). It is now possible to generate hierarchically porous nanostructures for various metals, including Pt, Pd, Au, Ag, Cu, Ni, and their alloys. To aid a better understanding of these synthetic methodologies and their fundamental principles, the systematic and comprehensive analysis of nanoporous metal fabrication, physicochemical properties, advanced functions, and corresponding applications are necessary.

**Table 1:** Synthesis strategies of hierarchically porous metals with different features and applications

Synthesis strategies	Hierarchical porosity	Morphologies	Compositions	Applications
Hard template	70–100 nm, 1–2 nm	3D nanowire network	Pt^[^ ^16^ ^]^	Cyclic voltammetry^[^ ^17^, ^18^ ^]^
	20–100 nm, 4–6 nm	3D nanowire network	Pt, Pd^[^ ^17^ ^]^	
Soft template	≈50 µm, ≈500 nm	3D monoliths	Ag^[^ ^19^ ^]^	
Hard‐soft co‐templates	≈460 nm, 3–4 nm	2D thin film	Pt^[^ ^18^ ^]^	
	≈45 nm, ≈4 nm	2D membrane	Pt^[^ ^20^ ^]^	
Direct dealloying	≈50 nm, ≈3 nm	2D foils	PtFe^[^ ^21^ ^]^	Formic acid oxidation,^[^ ^22^, ^23^, ^24^ ^]^ Methanol oxidation^[^ ^21^, ^25^ ^]^ Hydrogen evolution reaction^[^ ^26^ ^]^ Supercapacitors^[^ ^27^, ^28^ ^]^ Change transport^[^ ^29^ ^]^ Oxygen reduction reaction^[^ ^30^, ^31^ ^]^ Li‐O_2_ battery^[^ ^32^ ^]^ Oxidation reaction^[^ ^28^ ^]^ Electrochemical glucose sensing^[^ ^24^ ^]^
	≈50 nm, ≈5 nm	3D network	Pd^[^ ^23^ ^]^	
	30–350 nm, 25–70 nm	3D bulk	Cu^[^ ^33^ ^]^	
Two‐step dealloying	1–2 µm, ≈8 nm	2D membrane	Au^[^ ^34^ ^]^	
	≈100 nm, ≈3.5 nm	3D monolith	PtAu^[^ ^22^ ^]^	
	≈1 µm, ≈100 nm	2D film	Au^[^ ^25^ ^]^	
	≈1 µm, ≈10 nm	3D network	CuTi^[^ ^26^ ^]^	
	≈185 nm, ≈6 nm	3D network	NiCuMn^[^ ^27^ ^]^	
	≈200 nm, ≈15 nm	3D nested‐network	Au^[^ ^29^ ^]^	
	≈1000 nm, ≈ 5 nm	3D network	PtTi^[^ ^30^ ^]^	
	≈70 nm, ≈6 nm	3D network	PtCu^[^ ^31^ ^]^	
	80–100 nm, 5–20 nm	3D network	Au^[^ ^32^ ^]^	
Dealloying‐based methods	10–1000 µm, 30–500 nm	3D monolith	Au^[^ ^28^ ^]^	
	50–60 nm, 4–5 nm	3D network	PdCu^[^ ^24^ ^]^	
	≈100 nm, ≈10 nm	2D ribbon	Au^[^ ^35^ ^]^	
Hard template and dealloying	≈10 µm, 10–100 nm	0D hollow nanoparticle	Au^[^ ^36^ ^]^	H_2_O_2_ reduction reaction^[^ ^37^ ^]^ Oxygen reduction reaction^[^ ^37^ ^]^ Surface‐enhanced Raman scattering^[^ ^38^ ^]^ Methanol oxidation^[^ ^39^, ^40^ ^]^
	100–190 nm, 5–35 nm	1D microwire	Au^[^ ^37^ ^]^	
	≈200 nm, ≈3 nm	1D microtube	Cu^[^ ^38^ ^]^	
	≈100 nm, ≈10 nm	1D nanotube	Au, Au@Pt^[^ ^39^ ^]^	
Soft template and dealloying	≈15 nm, ≈2 nm	0D hollow nanoparticle	PtPd^[^ ^40^ ^]^	

**Figure 1:**
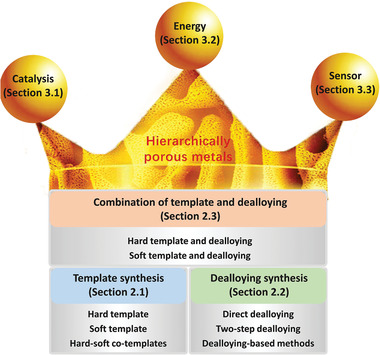
Diagram outlining the synthetic strategies and role of hierarchy in structure‐function for various applications of hierarchically porous metals.

Recently, a number of reviews on the design and synthesis of hierarchically porous nanostructures have been published, on materials such as zeolites,^[^ ^41^, ^42^ ^]^ metal oxides,^[^ ^43^, ^44^ ^]^ polymers,^[^ ^3^, ^45^ ^]^ carbons,^[^ ^46^, ^47^ ^]^ inorganic–organic hybrids^[^ ^48^, ^49^ ^]^ and single porous metals.^[^ ^50^, ^51^, ^52^, ^53^ ^]^ Some general synthetic strategies, such as wet chemistry and physical assistance, have been thoroughly studied and clearly described. However, the growth of nanocrystals and the formation of hierarchical pores constitute a very big difference between metals and the other materials mentioned above, and to our knowledge, there is no review on emerging metallic materials with hierarchically porous structures. An overview of synthetic strategies for introducing/controlling hierarchical porosity in metallic materials, and the resulting applications of these materials, therefore seems warranted. This review summarizes the most commonly used synthesis strategies (Section 2, mainly including templating and dealloying) to produce hierarchically porous metals and discusses how this hierarchy impacts on structure–function for applications in catalysis, energy, and sensing (Section 3) (**Figure** **1**). It is noted that this review is not intended to be comprehensive but intends to discuss the design and control of hierarchically porous metals referring to numerous published investigations. Finally, the current challenges and future prospects in this field are discussed.

## 2 Strategies to Synthesize Hierarchically Porous Metals

### 2.1 Template Synthesis

**Figure 2:**
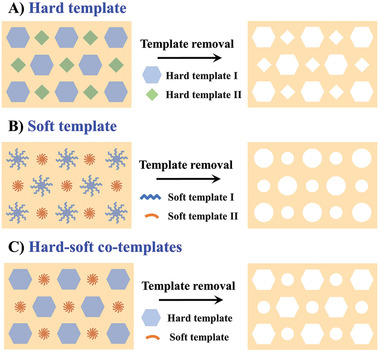
Schematic illustration of the three different template methods for synthesis of hierarchically porous metals: A) hard template, B) soft template, and C) hard‐soft co‐templates.

Template synthesis is an effective method for fabricating hierarchically porous metals with pore sizes ranging from micropores to mesopores to macropores. For example, mesoporous metals can be generated by depositing into the hexagonally packed cylindrical pore structures constructed by liquid crystal surfactants, while macroporous metals can be obtained by depositing into templates with larger‐size structures such as block copolymers, colloidal crystals, or anodic porous alumina.^[^ ^50^, ^54^, ^55^ ^]^ Benefiting from the directing shape/cooperation effect of the used templates, hierarchically porous metals synthesized by templating usually have a well‐defined shape, morphology, and spatial arrangement. The general processes of template synthesis are the cooperation of metal precursor and the template, removal of the template, and generation of nanostructured metals. For hierarchically porous metals, this templating process includes: i) preparation of an appropriate template with bimodal pore structures or dual/ternary templates with different pore sizes; ii) filtration of the void/pore spaces of pre‐prepared templates with suitable metal precursors by infiltration; iii) reduction of the corresponding metal precursors via chemical or electrochemical treatment; and iv) removal of the template to generate hierarchically porous metals. The templates used in chemical synthesis of hierarchically porous metals can be separated into three types: hard, soft, and combined templates (**Figure** **2**).

#### 2.1.1 Hard Template

The hard template method for the fabrication of porous materials can accurately replicate the structure of a template, which directly delivers the inverse replica of the original template.^[^ ^56^, ^57^, ^58^, ^59^, ^60^ ^]^ For hard template synthesis of hierarchically porous metals, at least two types of templates with different structures are necessary.

**Figure 3:**
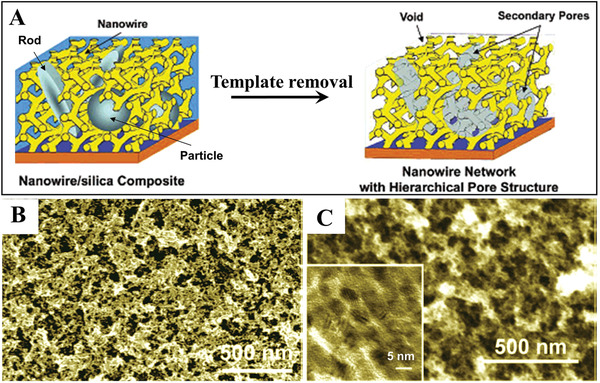
A) Schematic illustration of fabrication of a Pt nanowire network thin film with hierarchically controllable pore structure. B,C) SEM images of the as‐prepared Pt nanowire network thin film. Reproduced with permission.^[^ ^16^ ^]^ Copyright 2006, American Chemical Society.

Pt nanowire networks with hierarchical pore structures have been synthesized by using mesoporous silica and colloidal silica particles as dual templates.^[^ ^16^ ^]^ The generation of both macropores and mesopores is realized by introducing large‐sized silica particles into the original mesoporous silica template. As shown in **Figure** **3**A, the dual templates consisting of mesoporous silica (acting as a primary porous template) and silica particles/rods (acting as a secondary porous template) were prepared in a first step. Then, the mesoporous channels were fully filled with Pt by continuous electrodeposition. Finally, Pt nanowire networks with meso/macroporous structures were produced by synchronous removal of the dual templates (Figure 3B,C). The diameters and mesostructures of the Pt nanowires can be easily controlled by tuning the diameter of mesopores (2‐20 nm) and mesostructures (including 2D wire‐like and 3D mesh‐like structures) of the primary mesoporous silica template. The large pores in the Pt networks can also be controlled by tuning the sizes (20–30 nm) and shapes (e.g., spheres, rods) of the secondary pores of the silica particles. One distinguishing feature of this method is that pore sizes on different length scales can be independently controlled by the sizes of the primary and secondary pores.

#### 2.1.2 Soft Template

Soft templating is a common method for the synthesis of materials with ordered mesopores.^[^ ^61^, ^62^, ^63^, ^64^, ^65^, ^66^ ^]^ Compared to hard templates composed of rigid or semirigid materials, the building blocks of soft templates form via self‐assembly of surfactants micelles. Through coassembling with these blocks, metal precursors can be well‐dispersed to fabricate a certain nanostructure. After reduction and removal of surfactants, porous mesostructured metals are obtained. This is a big difference between reducing formation of metals and hydrolysis‐condensation formation of other sol‐gel materials. Therefore, it is not easy to generate bimodal pores by only using soft templates, even using dual soft templates. It has to be pointed out that the porous structures formed by soft templates are often not well ordered.

**Figure 4:**
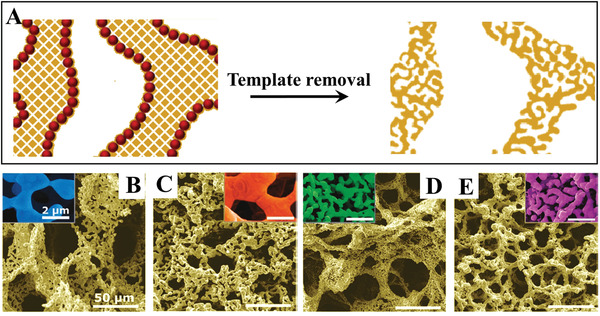
A) Schematic illustration of a nanocasting approach to hierarchically porous Ag. B–E) SEM images of hierarchically porous Ag monolith with various pore sizes. Reproduced with permission.^[^ ^19^ ^]^ Copyright 2011, American Chemical Society.

The rational design of dual templates can be considered as a breakthrough in the synthesis of hierarchically porous metals. For example, a nanoporous cross‐linked polymer template method was developed by Lee and Mohraz to prepare hierarchically porous silver monoliths with bicontinuous architectures.^[^ ^19^ ^]^ The polymer template, namely bicontinuous interfacially jammed emulsion gels (bijels), consisted of two continuous, incompatible fluid phases, that were chosen as soft templates for novel hierarchically porous metals. As shown in **Figure** **4**A, a macroporous polymer monolith with a nanoporous polymer phase was first formed from the colloidal bijel. Then, the surface particles were etched, and silver was deposited into the polymer phase by reducing silver ions in aqueous solution. The whole polymer template was finally removed by pyrolysis while sintering the silver nanoparticles, leading to the generation of a hierarchically porous Ag monolith (Figure 4B–E). The pore channel diameters can be tuned independently while maintaining the 3D spinodal morphology. It is notable that the available pore sizes can span almost 4 orders of magnitude, from tens of nanometers to over a hundred micrometers, highlighting the versatile nature of this method.

#### 2.1.3 Soft–Hard Co‐Templates

The combination of soft and hard templates not only enables the efficient synthesis of metal nanoarchitectures with hierarchically porous structures but also regulates the shapes and structures of these porous metals.

**Figure 5:**
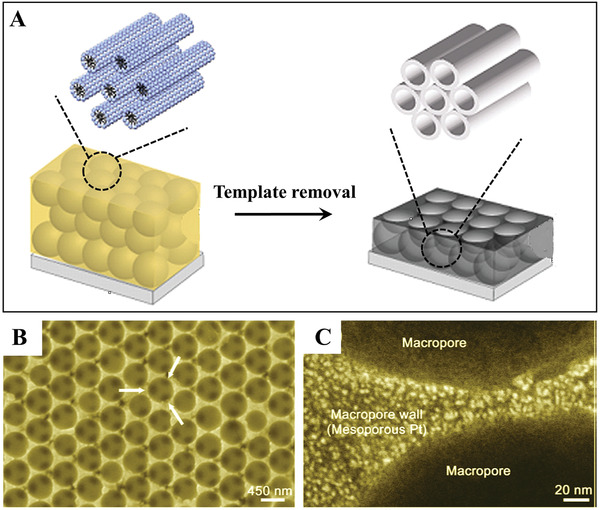
A) Schematic illustration of the fabrication process for preparing a hierarchically porous Pt film. B,C) SEM images of the as‐prepared hierarchically porous Pt film. Reproduced with permission.^[^ ^18^ ^]^ Copyright 2006, Elsevier.

Well‐defined Pt thin films with a macro–meso bimodal pore system have been fabricated by combining the modified liquid crystal template method and a colloidal crystal template.^[^ ^18^ ^]^ As shown in **Figure** **5**A, polystyrene (PS) spheres with a close‐packed arrangement and a long‐range hexagonal order were first assembled onto an Au‐coated Si substrate through a dip‐coating method. Then, a pre‐prepared precursor solution for lyotropic liquid crystals (LLC) was added into the confined space among the PS spheres by capillary action. During the preferential evaporation of volatile solvent from the precursor solution, LLC were gradually grown. Finally, the Pt thin films with a well‐defined hierarchical macro‐meso pore system were obtained by deposition of Pt in the presence of LLC and removal of both the surfactant and PS spheres (Figure 5B,C). The high‐quality PS sphere template is crucial to the resulting product, which depends on the ordering of this template. Moreover, this synthesis process could be further associated with lithography techniques to create multimodal porous materials with tunable compositions.

**Figure 6:**
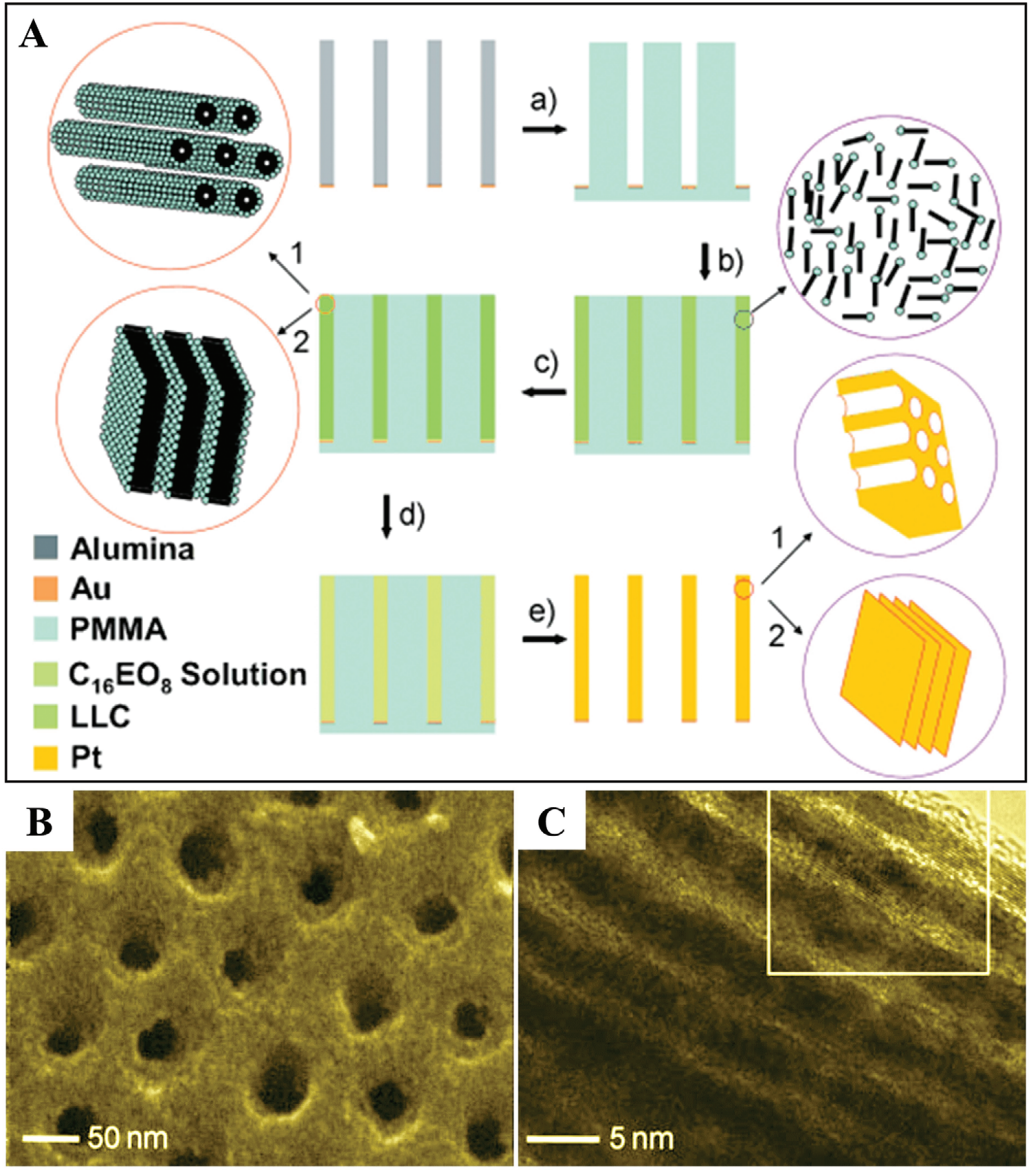
A) Schematic illustration of the preparation of hierarchically porous Pt membranes. B,C) SEM images of the as‐prepared hierarchically porous Pt membranes. Reproduced with permission.^[^ ^20^ ^]^ Copyright 2010, Wiley‐VCH.

Ordered metal nanostructures with hierarchical porosity can also be fabricated by rational utilization of combined templating techniques. A novel two‐step method using octaethylene glycol monohexadecyl ether (C_16_EO_8_) as a LLC former and reverse porous poly(methyl methacrylate) (PMMA) cast from porous anodic alumina (PAA) as a hard template has been reported by Webley et al. for the synthesis of hierarchically porous Pt membranes.^[^ ^20^ ^]^ As illustrated in **Figure** **6**A, the PMMA template was first prepared from a porous anodic alumina mold. Then, C_16_EO_8_ solution was injected into the nanoscale void space of the PMMA template. When the temperature was below the LLC melting point, the micellar solution was transformed into an LLC. Subsequently, Pt was deposited into the nanoscale voids of the LLC, which served as a structure‐directing medium in the production of Pt mesoporous nanostructures. After removal of the PMMA and LLC, ordered hierarchical porous Pt membranes were obtained (Figure 6B,C). In this method, the Pt deposition rate is relatively slow due to the low diffusion rate of Pt ions in the nanoscale voids of the LLC. Moreover, the mesostructures and macroscopic sizes can be easily tailored by changing the concentration of C_16_EO_8_ and nature of the porous anodic alumina template.

### 2.2 Dealloying Synthesis

Dealloying, namely selective dissolution of one or more active components from a solid alloy/compound (consisting of both active and inactive components to create a porous residue) has been explored as a versatile way to prepare porous metals with variable compositions and sizes.^[^ ^67^, ^68^, ^69^, ^70^, ^71^, ^72^ ^]^ Based on the corrosion conditions, dealloying can be divided into chemical dealloying (in acid/base solutions) and electrochemical dealloying (in electrochemical systems). With increased understanding of the pore formation mechanism by the growth of nanotechnology and nanoscience at the beginning of 21st century, some dealloying methods have been developed for preparing hierarchically porous structures of Au, Pd, Cu, PtAu, PtCu, PtFe, etc. via the dealloying of alloy systems. Generally, according to their dealloying step, there are three strategies for preparing hierarchically porous metals including direct dealloying, two‐step dealloying and dealloying‐based methods.

#### 2.2.1 Direct Dealloying

A simple dealloying method to hierarchical metals is the selective removal of sacrificial phases of a metallic composite. Technologically, a composite with various sacrificial phases and/or components enables the fabrication of a hierarchical metal with various porosities. Selective dealloying allows the porosity and pore size to be varied by changing the starting phase ratio, particle diameter, and treatment method. The porosity and the pore size therefore can be tuned over a wide range. Adjusting the initial volume fraction of the sacrificial phase has a corresponding effect on the porosity.

**Figure 7:**
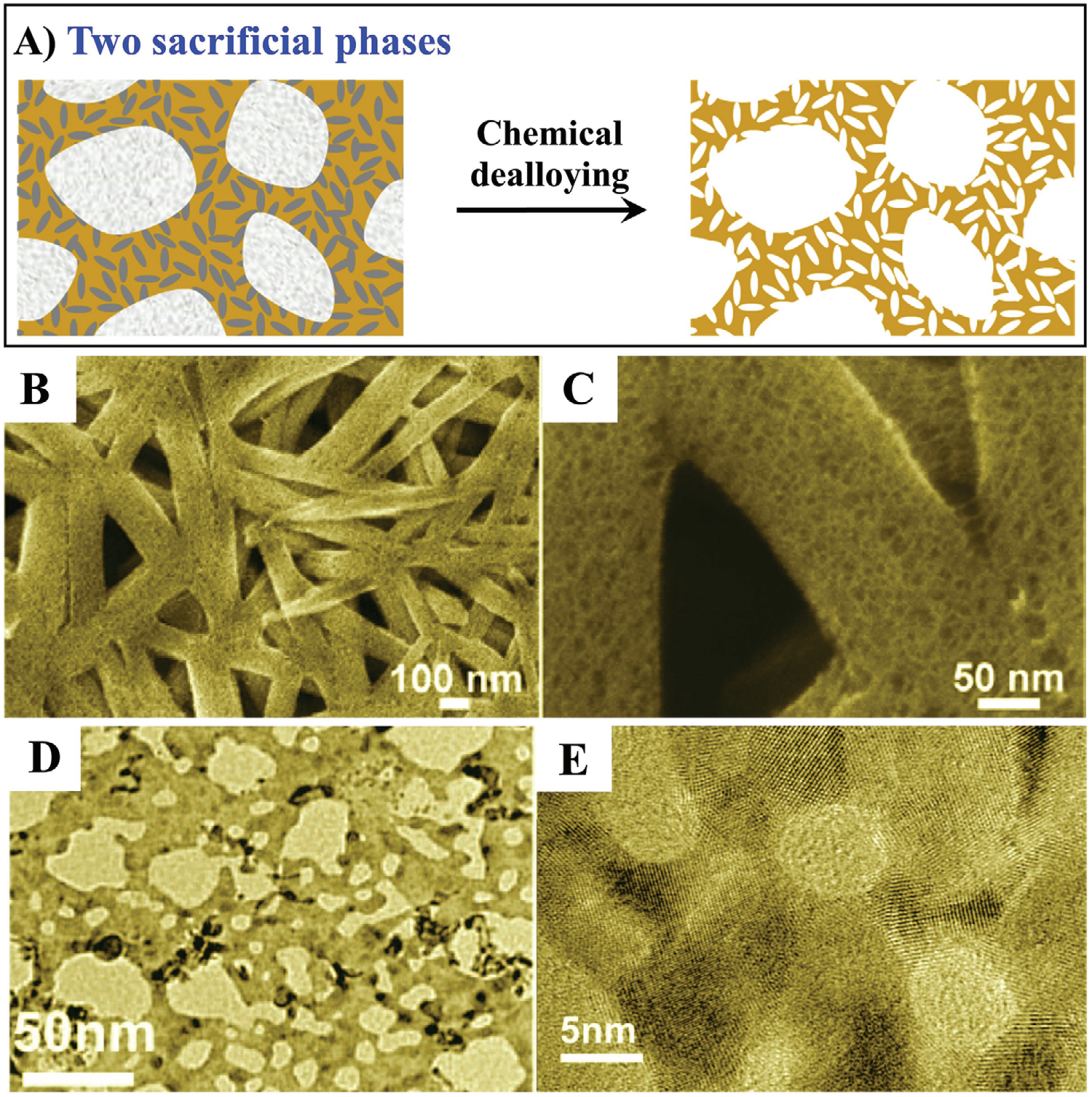
A) General strategy for hierarchical pore fabrication via direct dealloying. B,C) SEM images of a hierarchically porous PtFe alloy. Reproduced with permission.^[^ ^21^ ^]^ Copyright 2012, American Chemical Society. D,E) SEM images of hierarchically porous Pd. Reproduced with permission.^[^ ^23^ ^]^ Copyright 2008, American Chemical Society.

A composite of PtFeAl, as an example, is chosen to help to understand this process (**Figure** **7**A).^[^ ^21^ ^]^ Dealloying of Al was conducted in 2.0 m NaOH solution for 48 h at room temperature because Al can be selectively removed while Pt and Fe are stable under these conditions. The resulting PtFe alloys are composed of many interconnected large ligaments on the order of hundreds of nanometers, with these ligaments further consisting of 3D networks with typical sizes of around 3 nm (Figure 7B,C). It is found that the specific two‐phase structure of the PtFeAl precursor alloy is responsible for the production of the hierarchically nanoporous structure, in which the dissolution of segregated Al generates the larger pores and ligaments while smaller pores/ligaments result from dealloying of Al in the residual PtFeAl alloy. However, this approach is limited to metal systems with two‐phase precursors.

Moreover, hierarchically nanoporous metals can also be fabricated by simultaneous dealloying of two sacrificial components in one phase. Chen et al. reported the fabrication of hierarchically nanoporous Pd by electrochemical dealloying of multicomponent metallic glasses.^[^ ^23^ ^]^ Glassy Pd_30_Ni_50_P_20_ ribbons were first fabricated by single‐roller melt‐spinning in vacuum, followed by selective dissolution of the Ni and P in glassy Pd_30_Ni_50_P_20_ in a sulfuric acid solution. Since the standard electrode potential of P is more negative than that of Ni, P was dissolved faster than Ni. The ligaments in the primary porous structure were produced by the removal of P, which caused the nanocrystallization of the metallic elements to form a nanocrystalline PdNi solid solution. With further dealloying, the residual Ni in the nanocrystalline PdNi was then selectively dissolved, resulting in the generation of small secondary nanopores in the ligaments. Thus, by utilization of the different dissolution rates of P and Ni in a ternary alloy, the hierarchically nanoporous structure was formed via a one‐step direct electrochemical dealloying method (Figure 7D,E), indicating the versatility of composition design in a multicomponent alloy for producing various hierarchically porous metals with complex structures. Moreover, metallic glasses with a relatively wide range of components create a unique system for producing numerous hierarchically nanoporous metals which cannot be obtained from conventional metallic alloys.

#### 2.2.2 Dealloying/Annealing/Redealloying

Considering complex fusions of two or more sacrificial phases of particular metals, a dealloying/annealing/redealloying approach (so‐called two‐step dealloying) has been developed to synthesize hierarchically structured metals with two uniform pores using only one sacrificial phase.

**Figure 8:**
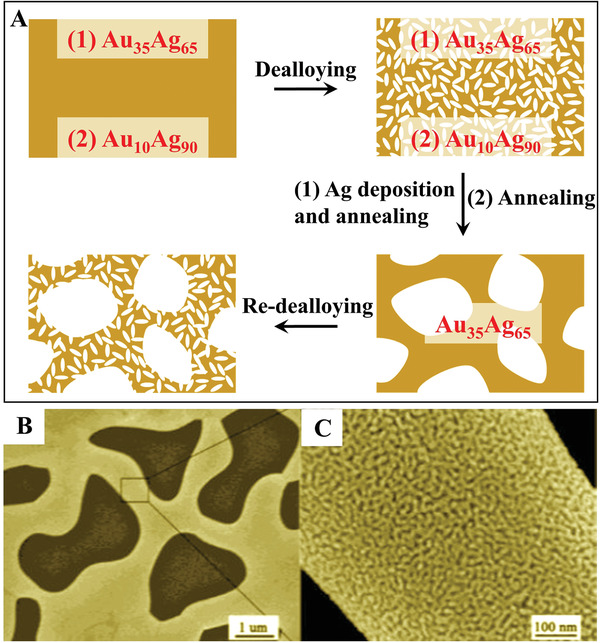
A) Two general strategies for hierarchical pore fabrication via two‐step dealloying. B,C) SEM images of hierarchically nanoporous Au. Reproduced with permission.^[^ ^34^ ^]^ Copyright 2003, American Chemical Society.

Nanoporous Au with bimodal pores is the most typical material fabricated by two‐step dealloying of AuAg alloys.^[^ ^29^, ^32^, ^34^ ^]^ The two general strategies in the synthesis of hierarchically nanoporous Au are illustrated in **Figure** **8**A. For the first strategy, a homogenous Au_35_Ag_65_ leaf was prepared by a wet‐chemical method. Then, regular etching of the pre‐prepared Au_35_Ag_65_ leaf was conducted under corrosion‐free conditions (immersion in a concentrated nitric acid solution, in which Ag is soluble but Au is stable). As a result, AuAg alloys with a unimodal pore size of 200–300 nm and Ag content of less than 5% were produced. Then, electrochemical deposition of an Ag layer onto the surface of the AuAg alloys was conducted, followed by annealing at a suitable temperature, generating Ag‐rich homogenized AuAg alloys with an increased pore size. Finally, the redealloying step removed the Ag elements to produce hierarchically porous Au. In the second strategy, first AuAg alloys with a much higher Ag content were prepared in order to avoid the complex Ag deposition process in the first strategy. The corrosion of this material generated a nanoporous AuAg alloy with an Ag‐rich composition, followed by thermal coarsening to create porous alloys with large pore sizes of ≈200 nm. The redealloying step generated hierarchically porous Au by completely dissolving the residual Ag elements to form nanopores inside the larger ligaments (Figure 8B,C).^[^ ^29^, ^34^, ^73^ ^]^

**Figure 9:**
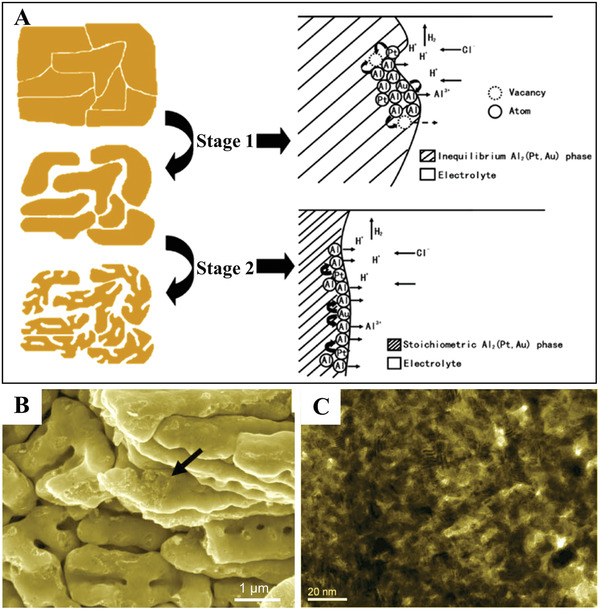
A) Schematic illustrations showing the pore formation process of the two‐stage electrochemical dealloying of the rapidly solidified Al_75_Pt_15_Au_10_ alloy in the neutral NaCl aqueous solution. B,C) SEM images of the hierarchically nanoporous PtAu alloy. Reproduced with permission.^[^ ^22^ ^]^ Copyright 2011, Royal Society of Chemistry.

Compared to the free corrosion phenomenon in chemical dealloying, electrochemical dealloying is more complicated.^[^ ^74^, ^75^, ^76^, ^77^ ^]^ Two important parameters should be considered. The first parameter is the parting limit, which indicates the limiting concentration of the inert component in an alloy for dealloying. The second parameter is the critical potential, which indicates the electrode potential threshold that has to be overcome in order to dissolve the less noble component from the alloy system. A hierarchically nanoporous PtAu alloy has been prepared by Zhang et al. via two‐step electrochemical dealloying of a ternary Al_75_Pt_15_Au_10_ precursor in a neutral sodium chloride solution (**Figure** **9**A).^[^ ^22^ ^]^ The Al_75_Pt_15_Au_10_ precursor consists of a single Al_2_(Pt,Au) phase with internal lattice vacancies. At the low potential of ‐0.4 V versus Ag/AgCl, the first‐step electrodealloying involves the partial dissolution of Al along with the disappearance of the lattice vacancies, resulting in the production of the stoichiometric Al_2_(Pt,Au). The second‐step electrodealloying at 0.6 V versus Ag/AgCl involves the complete dissolution of the residual Al together with the surface diffusion of Pt/Au, leading to the generation of the ultrafine nanoporous structure (Figure 9B,C).

#### 2.2.3 Dealloying‐Based Methods

Since dealloying is an effective method to produce a single porous nanostructure from alloys, hierarchically porous metals can be easily obtained by combining dealloying methods with other strategies of producing pores, such as 3D printing, galvanic replacement and rapid solidification.

##### 3D Printing and Dealloying

As an advanced fabrication technique, 3D printing has been increasingly applied to fabricate complex, multiscale architectures that break traditional scaling via digitally controlled deposition of phase‐change and reactive materials and solvent‐based inks.^[^ ^78^, ^79^, ^80^, ^81^, ^82^, ^83^ ^]^ Currently, it is easy to obtain 3D metals with macroscale‐ordered pores via 3D printing.^[^ ^84^, ^85^ ^]^ By combining 3D printing and dealloying methods, hierarchically porous metals with distinct bimodal pore structures could be synthesized.

**Figure 10:**
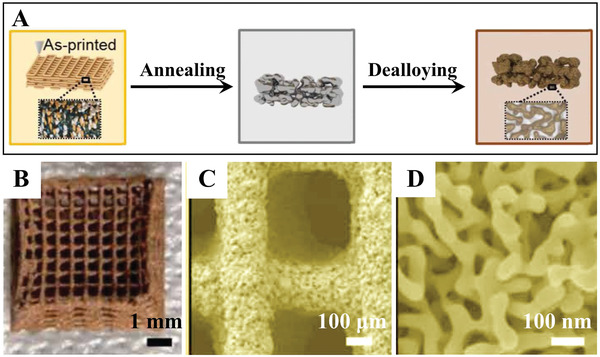
A) 3D printing of hierarchical nanoporous Au allows control over structure that spans over seven orders of magnitude in length scales, from centimeters to nanometers. B) Optical image and C,D) SEM images of hierarchically nanoporous Au. Reproduced with permission.^[^ ^28^ ^]^ Copyright 2018, AAAS.

3D‐printed hierarchically nanoporous Au with engineered nonrandom macroarchitectures has been reported by Biener et al. via combination of 3D printing and dealloying.^[^ ^28^ ^]^ The overall procedures involve three steps of printing, annealing and dealloying (**Figure** **10**A). A viscous, paste‐like ink made of a mixture of Ag and Au microparticles with a Ag/Au atomic ratio of 7/3 (the selection of this ratio is due to the production of stable nanoporous Au after dealloying of Ag) was first prepared as the raw material for preparing the ink. Under computer control, the prepared ink was deposited onto a planar substrate to create the predicted 3D porous architectures consisting of discontinuous Au and Ag particles bound by polymer. Then, the as‐printed architectures were annealed at 850 °C for 12 h in air to form a homogeneous AuAg alloy. Finally, the nanoscale porosity was produced to form a hierarchically porous structure by immersing the 3D AuAg alloy in a concentrated nitric acid solution to dissolve the Ag. The synthesized hierarchical porous Au displayed distinct structural length scales ranging from the digitally controlled microporous network structure (10–1000 µm) to the nanoscale pore/ligament morphology (30–500 nm) (Figure 10B–D). Although the 3D printing method is very attractive, the main drawback of this method compared to hierarchically porous methods is the very limited number of applicable alloy systems and the high input cost including hardware, feedstock, and maintenance.

##### Dealloying and Galvanic Replacement

Galvanic replacement offers an amazingly simple and versatile method for the fabrication of nanometals with hollow/porous structures.^[^ ^86^, ^87^, ^88^, ^89^, ^90^, ^91^ ^]^ This method involves the reduction of a relatively less active metal salt precursor by the core metal due to the redox potential difference between them, which often results in the generation of hollow/porous nanostructures with various morphologies, such as nanoboxes, nanocages, nanorings, and nanotubes.

A hierarchically nanoporous PdCu alloy has been synthesized by Liu et al. via the dealloying of a CuAl alloy and a subsequent galvanic replacement reaction with H_2_PdCl_4_ in aqueous solution.^[^ ^24^ ^]^ Nanoporous Cu (possessing a bicontinuous network structure with a typical ligament size of ≈50 nm) was prepared by dealloying the as‐prepared Cu_25_Al_75_ alloy foil in 1 m NaOH solution for 5 h at 30 °C. This was then used as a starting material and reducing agent to construct a hierarchically nanoporous PdCu alloy by placing it in H_2_PdCl_4_ aqueous solution under N_2_‐protected conditions at 5 °C for 2.5 h. The synthesized hierarchically nanoporous PdCu alloy exhibited a hollow tubular structure and a ligament shell composed of small pores and nanoparticles with a typical size of ≈4 nm.

##### Rapid Solidification and Dealloying

Rapid solidification is a mature and widely used process in the materials processing and manufacturing engineering industry. This has the salient advantage of retention of disordered crystalline structure in normally ordered materials and intermetallic compounds.^[^ ^92^, ^93^, ^94^, ^95^ ^]^ It is an effective method for the synthesis of two‐phase alloys with primary pores, which can be utilized as starting materials for the synthesis of hierarchically nanoporous metals by further dealloying of alloys.

Nanoporous Au ribbons with bimodal channel size distributions have been produced by Zhang et al. from AlAu alloys via combining rapid solidification and chemical dealloying.^[^ ^35^ ^]^ The AlAu precursor alloys, including the initial Al phase and the Al_2_Au intermetallic phase, were prepared via rapid solidification. During the rapid solidification, primary Al_2_Au dendrites precipitated, followed by the generation of the *α*‐Al solid solution phase from the residual liquid. In the process of etching, the fast excavation of the *α*‐Al phase out of the alloy contributed to the generation of large‐sized channels, and the dealloying of the Al_2_Au phase produced a nanoporous wall structure. In this work, the length scales of ligaments/channels in hierarchically nanoporous Au ribbons could be tuned by altering the dealloying solution and alloy composition.

### 2.3 Combination of Template and Dealloying

#### 2.3.1 Hard Template and Dealloying

As aforementioned, both hard templating and dealloying are very effective to produce nanoporous metals with precisely controlled structures. Thus, the combination of hard templates and dealloying methods allows the rational synthesis of hierarchically nanoporous metals with precise control of structure, morphology, and composition.

**Figure 11:**
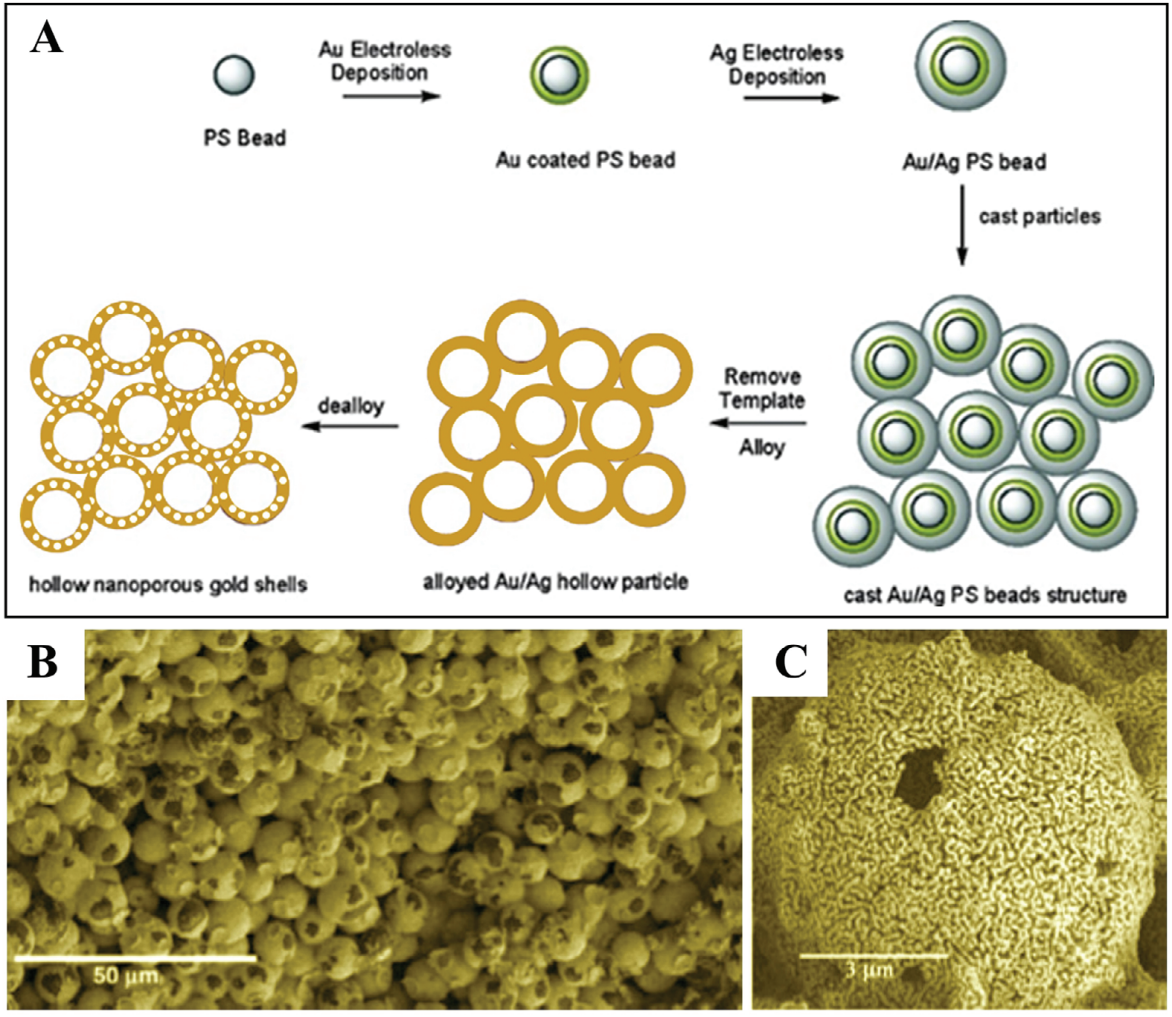
A) Schematic illustration of the synthesis of hollow nanoporous gold shells. B,C) SEM images of hierarchically nanoporous Au monoliths. Reproduced with permission.^[^ ^36^ ^]^ Copyright 2007, American Chemical Society.

Nyce et al. reported the synthesis of ultralow‐density gold monoliths with hierarchically nanoporous structures.^[^ ^36^ ^]^ Their strategy was to synthesize hollow Ag/Au alloys that could be dealloyed subsequently. As shown in **Figure** **11**A, PS beads were prepared as templates because of their advantages of facile synthesis and easy removal by heating. By stepwise electroless deposition of Au and Ag on PS spheres, Ag/Au PS core‐shell particles were obtained. Then, these isolated particles were assembled to form a monolith by casting processing, which is similar to the slip‐casting of ceramic and metallic particles where a suspension of Ag/Au PS particles was poured into a plaster of Paris mold. Hollow AgAu alloys were formed via removal of the PS template and alloying of Ag and Au by heating the monolith in an inert atmosphere at 400 °C. Finally, hierarchically nanoporous Au monoliths were obtained by selective dissolution of Ag from the AgAu alloys in a dilute nitric acid solution (Figure 11B,C).

#### 2.3.2 Soft Template and Dealloying

Recently, numerous nanoporous metals with various metals, such as Pt, Pd, PtPd, PtNi, AuPd, PtAu,^[^ ^96^, ^97^, ^98^, ^99^, ^100^ ^]^ have been synthesized by soft template methods via the self‐assembly of surfactants, which can easily be combined with other methods to fabricate hierarchically porous metals.

**Figure 12:**
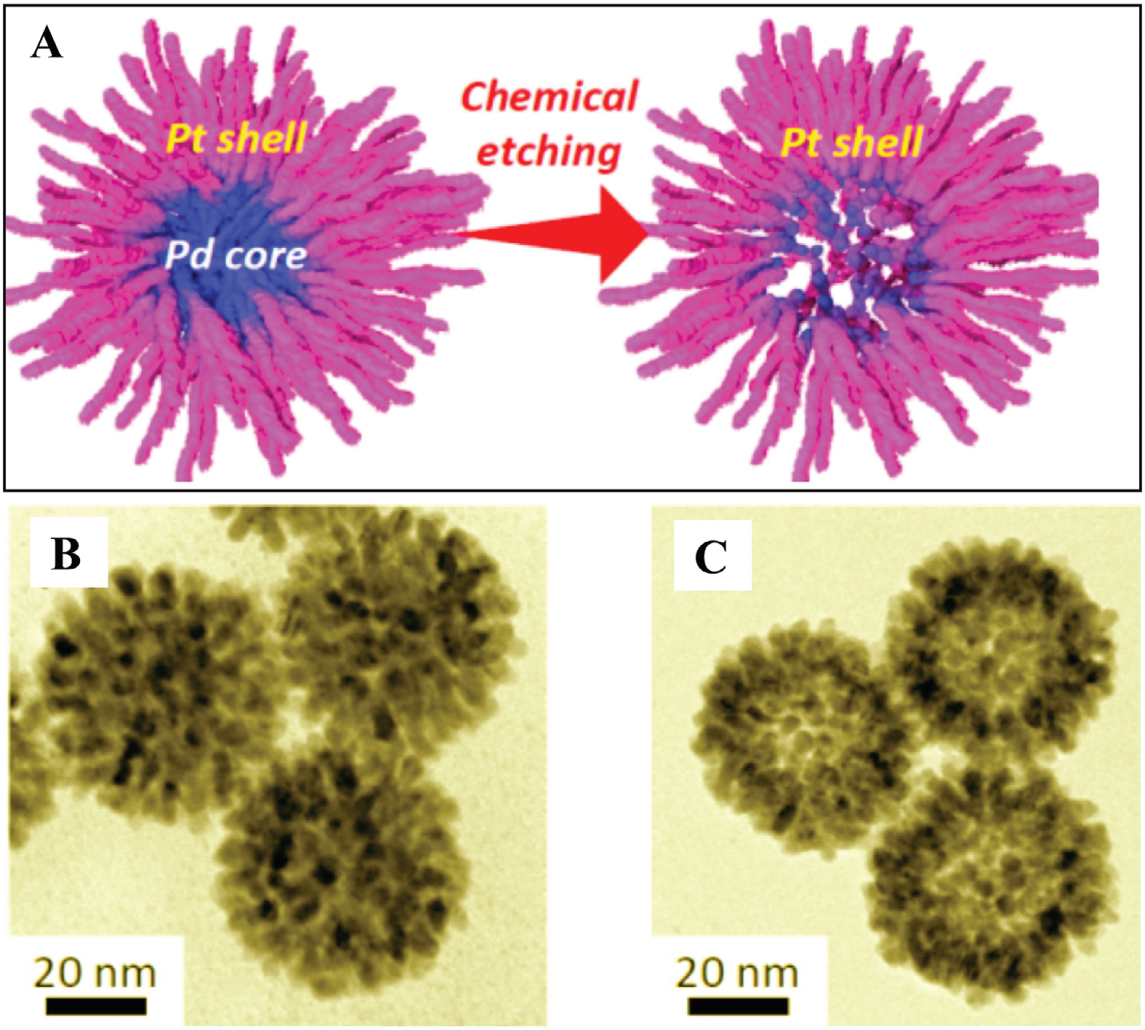
A) Schematic illustration of the formation of bimetallic dendritic nanocage with hollow interiors and porous dendritic walls. TEM images of dendritic Pt‐on‐Pd nanoparticles B) before and C) after chemical etching. Reproduced with permission.^[^ ^40^ ^]^ Copyright 2013, American Chemical Society.

Generally, hierarchically nanoporous metals fabricated by the combination of self‐assembly and dealloying have two steps: preparation of metal alloys with uniform nanoporous structures by the self‐assembly approach and then endowment of the nanoporous alloys with secondary pores by selectively dissolving the less‐stable component to form hierarchically nanoporous structures. For instance, Wang and Yamauchi prepared novel hierarchically nanoporous PtPd spheres with a hollow interior and a nanoporous exterior by this combined method.^[^ ^40^ ^]^ As shown in **Figure** **12**A, Pt‐on‐Pd spheres with nanoporous exteriors (Figure 12B) were first prepared in aqueous solution via self‐assembly by using Pluronic P123 as the structure‐directing agent and ascorbic acid as the reducing agent at room temperature. Then, the hierarchically nanoporous PtPd nanocages with hollow interiors and porous exteriors (Figure 12C) were obtained by selectively dissolving the excess Pd using concentrated nitric acid. This method is relatively facile and can be easily scaled up for routinely synthesizing hierarchically porous metallic nanocages.

## 3 Hierarchy for Structure–Function

Hierarchical porous structures are commonly found in living organisms and are of key importance to achieve optimal properties and performance in a range of contexts.^[^ ^11^, ^101^ ^]^ Hierarchy design in nanostructured metals has been used extensively to promote functions such as fast molecule transport by interconnected pores, rapid ion exchange via exposed sites and high electronic conductivity through interconnected networks.

### 3.1 Catalysis

#### 3.1.1 Oxygen Reduction Reaction

Many electrochemical energy devices, such as fuel cells and metal–air batteries, rely on the cathodic oxygen reduction reaction (ORR), in which oxygen molecules react with the protons/water and electrons to form H_2_O/OH^−^.^[^ ^102^ ^]^ However, the extremely sluggish ORR kinetics is currently hampering the extensive usage of these devices due to the consequential decrease in energy efficiency.^[^ ^103^ ^]^ Therefore, high‐efficient ORR catalysts are important to solve this problem.

**Figure 13:**
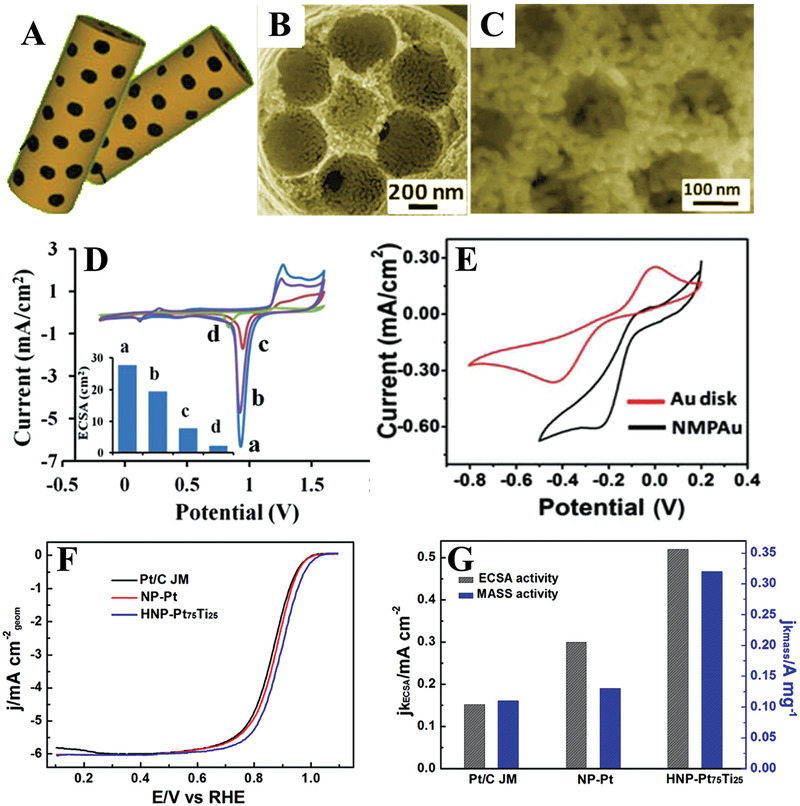
A) Schematic illustration and B,C) SEM images of hierarchically macro/mesoporous Au microwires. D) CVs of (a) hierarchically macro/mesoporous Au wires, (b) mesoporous Au microwires, (c) macroporous Au microwires, and (d) Au disk electrode in 0.5 m H_2_SO_4_ with a scan rate of 50 mV s^–1^; inset displays ECSA of the different electrodes. E) The electrocatalytic activity of the macro/mesoporous gold wires (black) and Au disk electrode (red): the O_2_ reduction in O_2_‐saturated 0.5M KOH solution (CV scan rate 50 mV s^–1^). Reproduced with permission.^[^ ^37^ ^]^ Copyright 2013, Royal Society of Chemistry. F) Polarization curves for the ORR on HNP‐Pt_75_Ti_25_, NP‐Pt, and Pt/C catalysts in the O_2_‐saturated 0.1 m HClO_4_ solution at room temperature at 1600 rpm, scan rate: 10 mV s^–1^. G) Specific kinetic and mass kinetic current densities at room temperature for HNP‐Pt_75_Ti_25_, NP‐Pt, and Pt/C catalysts at 0.90 V. Reproduced with permission.^[^ ^33^ ^]^ Copyright 2015, Elsevier.

Specifically, hierarchically porous alloys are a promising class of desirable electrocatalysts. Metal nanostructures with hierarchical pore systems show superior performance in ORR over metal nanostructures with single pore systems or other morphologies. For example, highly ordered hierarchical macro‐mesoporous Au microwires (**Figure** **13**A–C) have been synthesized based on coupling templating and dealloying approaches.^[^ ^37^ ^]^ The electrocatalytic surface area (ECSA) of the macro‐mesoporous Au microwires was 27.7 cm^2^ (Figure 13D); this was higher than that of mesoporous Au microwires (19.5 cm^2^), Au microwires (7.8 cm^2^), and Au disk electrodes (2.3 cm^2^), demonstrating that there is an increase in exposed active sites in hierarchically porous structures. Moreover, the macro‐mesoporous Au also showed an enhanced catalytic activity toward the ORR (Figure 13E). Due to the synergetic effect of macropores (associated with fast diffusion) and mesopores with high surface areas, the reactive molecules can easily enter the hierarchical pores and access active sites, hence enhancing electrocatalytic activity.

For noble metals, the introduction of less expensive metals to form bimetallic alloys is a good choice to further improve the practicality and activity by synergetic effects between each metal and reduce the consumption of scarce precious metals. For instance, hierarchically nanoporous PtTi bimetallic alloys with a bimodal size distribution and tunable element ratio have been prepared by a two‐step dealloying process.^[^ ^30^ ^]^ The as‐synthesized hierarchically nanoporous Pt_75_Ti_25_ alloys are composed of 3D continuously interconnected nanoporous structures with two different kinds of ligament/pore sizes of ≈1000 and ≈5 nm. In comparison to the nanoporous Pt and commercial Pt/C catalysts, hierarchically nanoporous Pt_75_Ti_25_ alloys displayed improved mass and specific activities in the ORR (Figure 13F,G), which can be described to the unique hierarchically porous structures and the alloying effect of Ti that can optimize the d‐band center of Pt.

#### 3.1.2 Methanol Oxidation Reaction

The methanol oxidation reaction (MOR) occurred at the anode in direct methanol fuel cells involves a multi‐step reaction process including the adsorption and oxidation of the methanol molecules.^[^ ^104^ ^]^ Currently, Pt has been demonstrated as the most efficient catalyst for MOR due to the suitable energy for the dissociative adsorption of methanol.^[^ ^105^ ^]^ However, Pt catalysts are highly susceptible to poisoning by surface‐adsorbed reaction intermediates (e.g., CO), leading to quick loss of the catalytic activity.^[^ ^106^ ^]^ Development of Pt‐based catalysts with high catalytic activity and stability is wisdom to tackle this CO poisoning problem.

Introduction of pore structures in catalysts is an effective approach for fast and effective transport in MOR due to the high surface area. Particularly, dendritic architecture with a hierarchically porous structure possesses numerous advantageous features such as high surface‐to‐volume ratio, high surface roughness, and lots of active edges and tips. For example, PtPd nanoparticles with a hollow interior and porous dendritic shell have been prepared via selective chemical etching of Pd cores from dendritic Pt‐on‐Pd nanoparticles.^[^ ^40^ ^]^ The as‐synthesized dendritic PtPd hollow nanoparticles displayed largely enhanced activity as well as stability compared to dendritic PtPd nanoparticles toward MOR, which can be attributed to the sufficient accessible active sites at both interior–exterior surfaces and the spatial and local separation of Pt nanoarms.

#### 3.1.3 Other Reactions

Recent researches have emerged more prosperous of many catalytic reactions, such as CO_2_ reduction, hydrogen evolution, formic acid oxidation, and etc.^[^ ^107^, ^108^, ^109^, ^110^ ^]^ Since hierarchically porous metal catalysts possess unique characteristics of high activity, high selectivity, and high stability in comparison with their bulk counterparts, investigations of hierarchically porous metal catalysts for these reactions have attracted much attention.^[^ ^22^, ^23^, ^24^, ^26^, ^111^ ^]^

**Figure 14:**
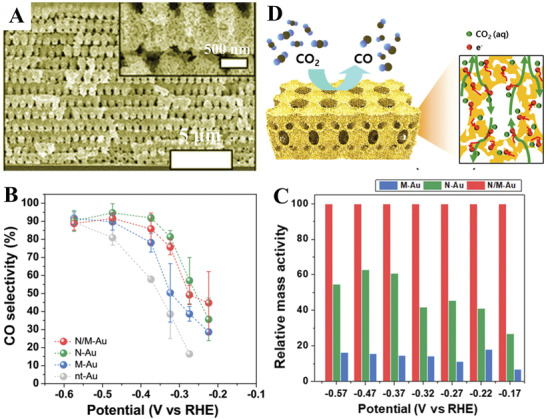
A) SEM images of hierarchically porous Au. B) CO selectivity of various Au nanostructures for electrocatalytic CO_2_ reduction. C) Relative mass activity comparing between various Au nanostructures under different potential. D) Schematic illustration (left) and the cross‐sectional view with the expected reaction pathway (right) for hierarchically porous Au electrode. Reproduced with permission.^[^ ^111^ ^]^ Copyright 2020, National Academy of Sciences.

A 3D hierarchically porous Au (N/M‐Au, **Figure** **14**A) with nanopores (around 10 nm) and interconnected macroporous channels (200–300 nm) was fabricated by Hyun et al. via proximity‐field nanopatterning.^[^ ^111^ ^]^ Compared to nanoporous Au (N‐Au) and macroporous Au (M‐Au), N/M‐Au consisted of both nanopores and macropores displayed higher selectivity and mass activity over the whole potential region (Figure 14B,C). In this case, the nanopores with a high‐index Au surface in N/M‐Au can be attributed to the high CO selectivity. Moreover, the interconnected macropores contribute a bubble‐growth site and high‐efficient transport pathways enabling the fast mass transport of CO_2_ (Figure 14D).

### 3.2 Energy

Metal nanostructures with open porous structures have displayed good performance and considerable promise for applications in energy storage and conversion.^[^ ^50^, ^51^, ^112^ ^]^ However, the successful implementation of metal nanostructures is still limited by several unpleasant difficulties originating from their underlying conversion chemistry, such as low electronic conductivity and structural instability.^113^ Along with electronic conductivity, ionic diffusion is another key role for an electrode material in energy storage and conversion applications, such as supercapacitors, sodium‐ion batteries, and lithium‐sulfur batteries.^[^ ^114^, ^115^, ^116^, ^117^ ^]^ Therefore, hierarchically porous structures with continuous metallic architectures for improved electronic/ionic conductivity and structural stability could show great promise.

**Figure 15:**
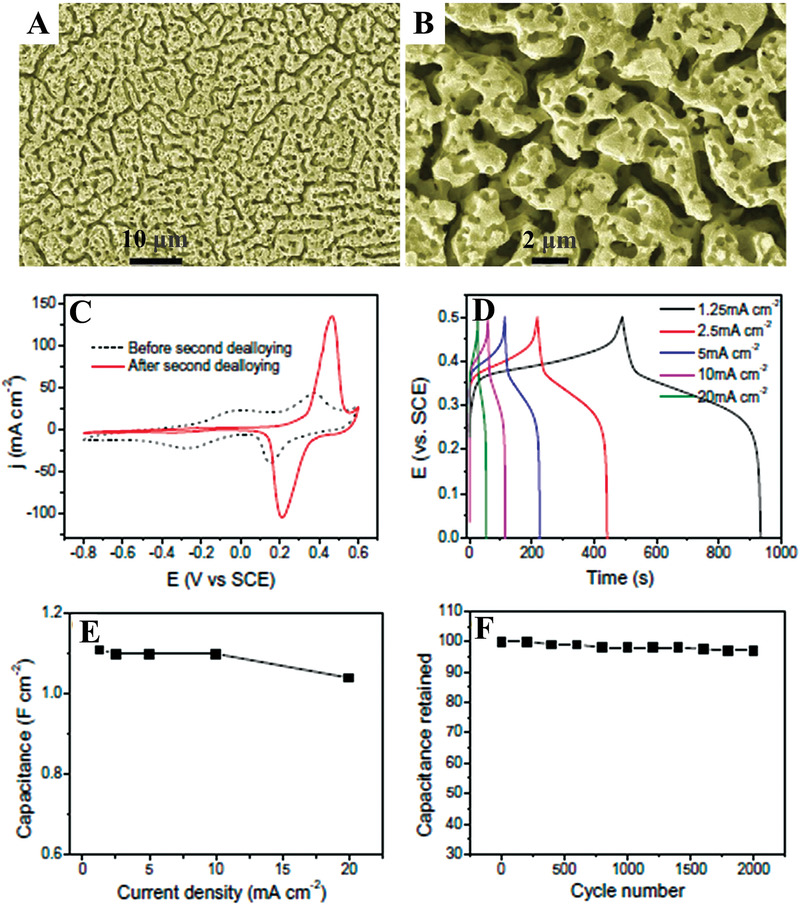
A,B) SEM images of a hierarchically nanoporous Ni alloy. C) CV curves of the annealed nanoporous Ni alloy before and after the second dealloying, scan rate: 40 mV s^–1^. D) Charge–discharge curves of the hierarchical electrode after the second dealloying. E) Rate dependence of the specific areal capacitance of the hierarchical electrode at different current densities and F) the capacitance retention as a function of cycle number at a constant charge‐discharge current density of 10 mA cm^–2^. The electrolyte is 1 m KOH aqueous solution. Reproduced with permission.^[^ ^27^ ^]^ Copyright 2014, Elsevier.

A hierarchically nanoporous Ni alloy (≈185 and ≈6 nm, **Figure** **15**A,B) was synthesized by Chen et al. via a two‐step dealloying approach.^[^ ^27^ ^]^ In comparison with the nanoporous alloy with large pore sizes obtained before the second dealloying process, the hierarchically porous Ni alloy displayed a much higher integration for the redox peaks (Figure 15C), indicating its larger charge storage due to its much higher surface area and small pores. When increasing the current density from 1.25 to 20 mA cm^–2^ (Figure 15D,E), the specific areal capacitance of the hierarchically nanoporous Ni alloy varied from 1.11 to 1.05 F cm^–2^ with ≈95% retention. This result demonstrates the high‐rate capacity performance of the hierarchically nanoporous Ni alloy in comparison with most reported pseudocapacitive electrodes, which can be ascribed to the high electron/ion conductivity resulting from the 3D continuously interconnected pore systems. Moreover, after 2000 cycles at the 10 mA cm^–2^ charge/discharge rate, ≈97% of capacitance remained (Figure 15F), indicating excellent cycling stability, which was attributed to the mechanical stability and fast electron/ion transport of the hierarchically nanoporous structures.

### 3.3 Sensor

Metallic materials with various structures, such as nanospheres, nanoshells, nanogaps, nanoholes etc., have been used as substrates for applications in chemical and biomolecular sensing.^[^ ^118^, ^119^, ^120^, ^121^, ^122^ ^]^ Among them, hierarchically porous metals not only show bimodal porous channels with large surface areas which contribute to the high accessibility of target molecules across a wide range of concentrations but also have a unique continuous metallic skeleton with high intrinsic conductivity and mechanical stability, resulting in their advantageous features of good signal reproducibility, high precision, and accuracy. Therefore, this type of metal can also be an ideal electrode material for electrochemical sensors.

**Figure 16:**
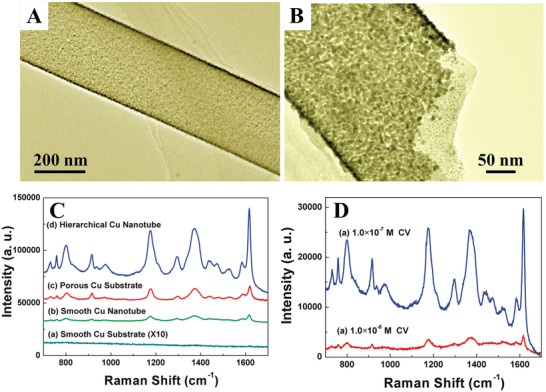
A,B) TEM images of hierarchical Cu nanotubes. C) Raman spectra of 1.0 × 10^–6^ m crystal violet adsorbed on different substrates. D) SERS spectra of different concentrations of crystal violet on hierarchical Cu microtubes: a) 1.0 × 10^–7^ m and b) 1.0 × 10^–8^ m. Reproduced with permission.^[^ ^38^ ^]^ Copyright 2010, American Chemical Society.

For instance, hierarchical nanostructured metals can greatly enhance surface‐enhanced Raman scattering (SERS) activity because of their rough surfaces originating from small pores and large surface‐to‐volume ratios which contribute to superior surface‐sensitive properties in SERS compared to metals with low dimensional structures. The SERS properties of hierarchically porous Cu nanotubes with evenly distributed nanoscale pores on the sidewalls (**Figure** **16**A,B) were investigated by using crystal violet as the analyte.^[^ ^38^ ^]^ Compared to porous Cu substrates, smooth Cu nanotubes, and smooth Cu substrates, hierarchically porous Cu nanotubes showed a much higher absolute Raman intensity in 1.0 × 10^–6^ m crystal violet solution (Figure 16C). This can be ascribed to the curved morphology of the sub‐microtubes, giving increased surface areas, evenly distributed nanopores and 1‐D geometry. When decreasing the concentration of crystal violet to as dilute as 1.0 × 10^–8^ m, an obvious SERS signal could also be detected using the hierarchically porous Cu nanotubes (Figure 16D), indicating the superior sensitivity of hierarchically porous structures as SERS substrates.

## 4 Conclusion and Perspectives

This review has summarized the common synthesis strategies for preparing hierarchically porous metals including template synthesis, dealloying synthesis, and the combination of template and dealloying. It is notable that most of these common strategies can be modified and/or extended to other metal systems, although for conciseness we only focused on a limited number of examples in the section on synthesis strategies. Moreover, hierarchical design in structure–function for applications in catalysis, energy and sensing have also been demonstrated, confirming that the synthesis of hierarchically porous metals is of great importance in both academia and industry.

Although rapid and significant development has been made in the synthesis of hierarchically porous metals, research in this field is still at the exploration stage and several issues need to be addressed, such as disordered porous structures, limited compositions, and undefined shapes and morphologies. In order to overcome these challenges and make steady and further progress, in our opinion, research directions on hierarchically porous metals should be developed as follows.

Firstly, more synthetic strategies for hierarchically porous metals should be developed. The present strategies are suitable for obtaining noble metals such as Au, Pt, Cu, but largely limit the component diversity of the resulting hierarchically porous metals. There is thus a great need to develop general approaches for the preparation of other hierarchically porous metals such as Fe, Co, Ni, and others. Moreover, the development of simple, efficient, and environmentally friendly strategies which can be performed at an industrial scale is required since the reported synthesis strategies to date all have at least one disadvantage, including multiple steps, long reaction times, high reaction temperatures, and so on. Since many hierarchically porous nanostructures of metal oxides/hydroxides have been already synthesized,^[^ ^1^, ^123^ ^]^ the directly thermal reduction of these metal oxides/hydroxides in the reductive atmosphere is supposed to be an efficient strategy for synthesizing corresponding hierarchically porous metals.

Secondly, more metals to form bi‐ or multimetallic alloys could be introduced. It is not easy to prepare multimetallic alloys especially in a particular morphology such as porous nanostructures because of the difference in redox potentials among different metals and the very harsh synthesis conditions that are needed. However, multicomponent alloys usually display superior performance compared to their fewer‐component counterparts due to synergetic effects, and more importantly, they exhibit fascinating properties and applications.^[^ ^124^, ^125^, ^126^ ^]^ Recently, high‐entropy alloys made of at least five metallic elements have attracted increasing interest in various applications due to their high entropy, lattice distortion, and cocktail effect.^[^ ^127^ ^]^ Creation of hierarchical pores in these high‐entropy alloys may be a promising method to produce hierarchically porous multimetallic alloys.

Thirdly, the pore structure of hierarchically porous metals should be precisely controlled. Fine‐tuning of the pore size and structure is of great importance in applications such as molecule selectivity in catalysis and host‐guest chemistry.^[^ ^128^, ^129^ ^]^ In addition, for hierarchically porous metals, the successful synthesis of highly ordered porous nanostructures is very attractive since hierarchically porous materials with ordered porous structures have been demonstrated as a class of materials with excellent properties. Characterization techniques are of great significance for understanding of the pore formation mechanism of hierarchically porous metals. Two main types of techniques have been widely used. One is electron microscopy technique, including scanning electron microscopy, transmission electron microscopy and atomic force microscopy. The other is pore size/structure measurement, such as gas adsorption for micro/mesopores, and mercury intrusion for macropores. In addition, low‐angle X‐ray diffraction analysis can also be exploited to measure the ordered porous nanostructures.^[^ ^130^ ^]^ The development of advanced characterization methods to in situ observe the structural evolution is highly desired, for example, in situ transmission electron microscopy, which could provide the information on the structure, composition, and morphology during the pore formation.

Fourthly, the morphology and shape of hierarchically porous metals should be precisely controlled. Numerous studies demonstrated that the physical and chemical properties of metals are highly correlated with their morphology and shape.^[^ ^131^, ^132^, ^133^ ^]^ Hierarchically porous metals with precisely controlled morphology and shape offer the best prospects for maximum performance toward a specific reaction. For example, metal catalysts with high‐index facets usually display markedly increased activities in comparison with these low‐index facets due to the high density of low‐coordinated atoms including steps, edges, and kinks, which can serve as catalytically active sites.^[^ ^134^ ^]^

Finally, the study of the applications of hierarchically porous metals should be expanded. Although hierarchically porous metals have been employed in the fields of catalysis, energy, and sensing, such materials should also display interesting properties in other fields because of their unique porous and hierarchical nanostructures. Moreover, with the development of synthetic strategies for producing hierarchically porous metals with more components and more morphologies, it is clear that researchers will seek to use such materials in emerging applications in the future such as optics and life science.

Despite significant progress in the synthesis of hierarchically porous metals, further persistent efforts should be made. With the continuous development of preparation methods and characterization techniques in materials science, it is expected that notable advancements in the synthetic strategies and advanced applications of hierarchically porous metals will be achieved in the near future.

## Conflict of Interest

The authors declare no conflict of interest.
